# Supplementary material for: Development of Polymersomes Co-Delivering Doxorubicin and Melittin to Overcome Multidrug Resistance
Source: Molecules. 2023 Jan 21;28(3):1087. doi: 10.3390/molecules28031087 (PMC9920864; doi:10.3390/molecules28031087)
Supplement: Supplementary file 1 [file molecules-28-01087-s001.zip › molecules-2153624-supplementary.pdf]

Supporting Materials

Development of Polymersomes Co-Delivering Doxorubicin and Melittin to Overcome Multidrug Resistance

*Eunkyung Han[1], Doyeon Kim[1], Youngheun Cho[1], Seonock Lee[2], Jungho Kim[2], Hyuncheol Kim[1,3] **

[1] Department of Chemical and Biomolecular Engineering, Sogang University, 35 Baekbeom-ro, Mapo-gu, Seoul 04107, Republic of Korea

[2] Department of Life Science, Sogang University, 35 Baekbeom-ro, Mapo-gu, Seoul 04107, Republic of Korea

[3] Department of Biomedical Engineering, Sogang University, 35 Baekbeom-ro, Mapo-gu, Seoul 04107, Republic of Korea

* Corresponding author: hyuncheol@u.sogang.ac.kr

† These authors contributed equally to this work.

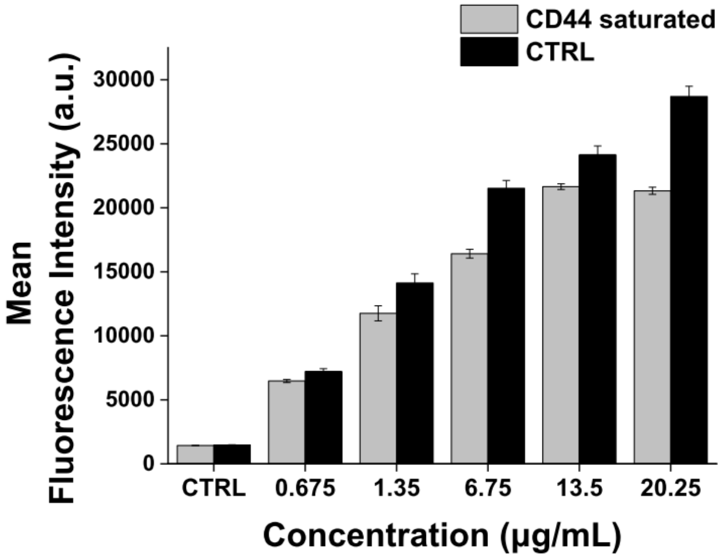

Figure S1. Flow cytometry for showing intracellular uptake of Dox-Mel PL in the MDR cells through the CD44 receptor. CD44 was blocked by using HA. Gray bar is for CD44 blocking cell expression, black bar is for normal cell expression. Also, indicated concentration is based on the Dox concentration.

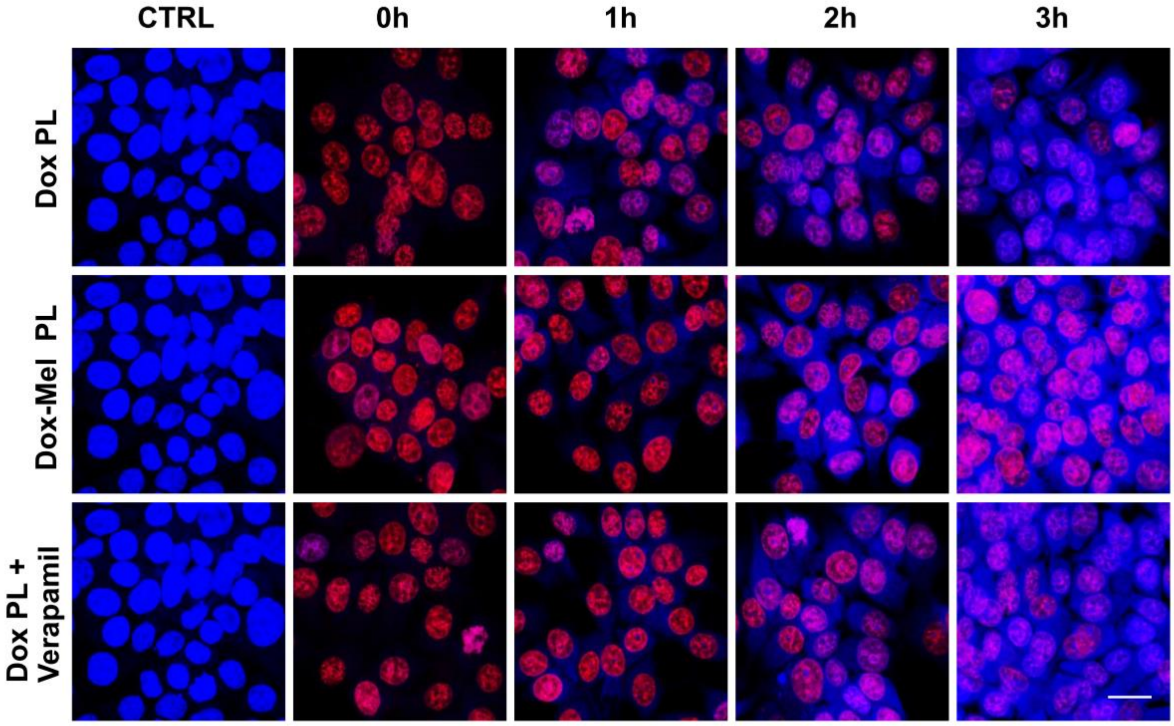

Figure S2. Intracellular retention of Dox. Confocal microscopy images showing Dox retention inside MCF-7 cells. The blue color represents cell nuclei. Red channel represents Dox (Scale bar=20μm)

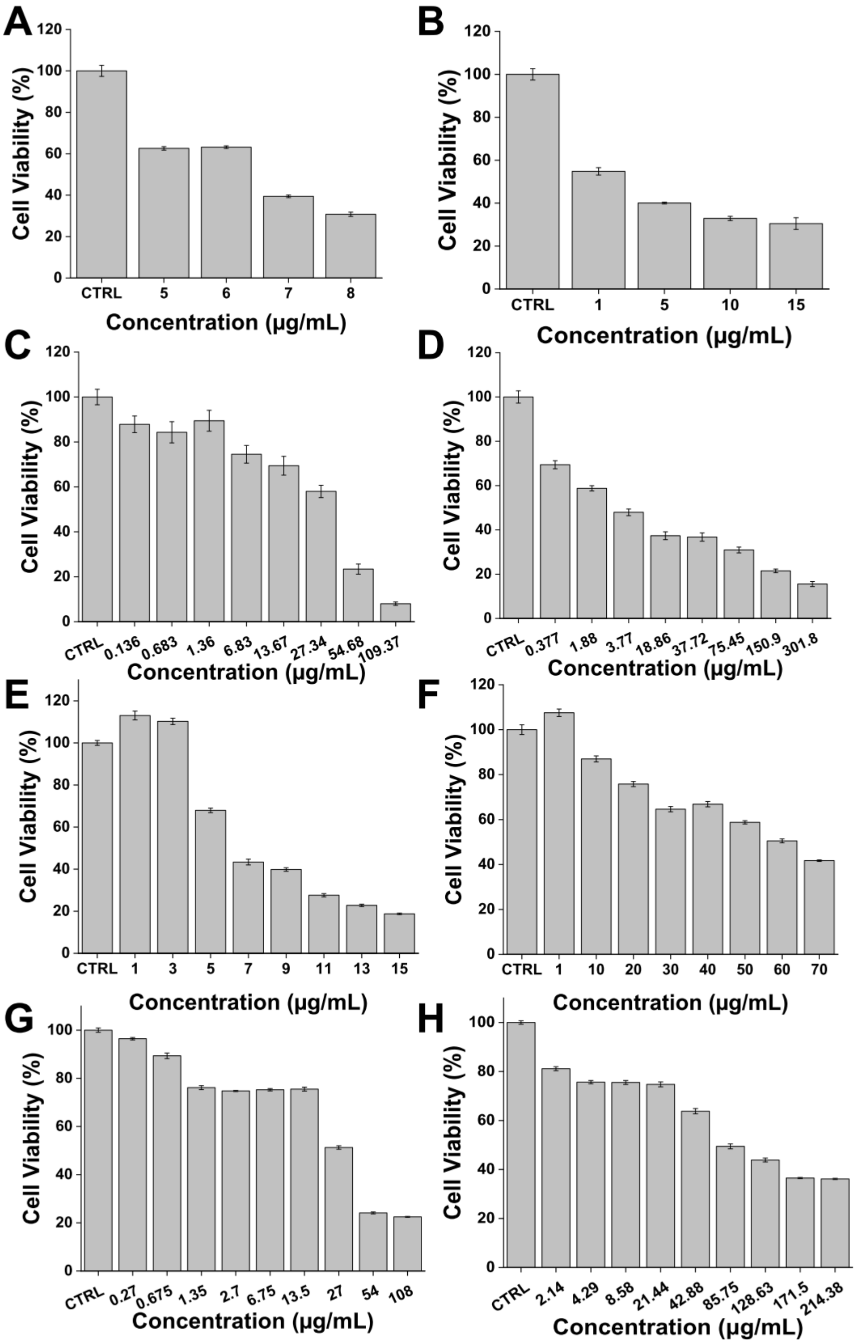

Figure S3. Cell Viability of free drugs and each PL-type in MCF-7 and MCF-7/ADR. (A) Treated with Free Mel in MCF-7 cell (B) Treated with Free Dox in MCF-7 cell (C) Treated with Mel PL in MCF-7 cell (D) Treated with Dox PL in MCF-7 cell (E) Treated with Free Mel in MCF-7/ADR cell (F) Treated with Free Dox in MCF-7/ADR cell (G) Treated with Mel PL in MCF-7/ADR cell (H) Treated with Dox PL in MCF-7/ADR cell
